# Upskilling, reskilling and deskilling when using welfare technologies in Swedish older adults’ care: A vignette-based qualitative study across four municipalities

**DOI:** 10.1016/j.ijnsa.2026.100665

**Published:** 2026-08-20

**Authors:** Susanne Frennert, Katrin Skagert

**Affiliations:** a Department of Design Sciences, Lund University, Lund, Sweden; b Department of Sociology and Work Science, University of Gothenburg, Sweden; c Division Digital Systems, Research Institutes of Sweden (RISE), Gothenburg, Sweden

**Keywords:** Welfare technology, Digitalisation, Home care, Nursing home, Vignette-based workshops, Skills development

## Abstract

**Background:**

In Swedish older adults’ care, welfare technology is often introduced to help address the pressures of population ageing and ongoing difficulties in recruiting and retaining staff. By welfare technology, we mean digital and technical solutions used in everyday care work with older adults, such as mobile documentation systems, digital door access, alarms and social robots. Earlier research suggests that opportunities for skills development are important for job satisfaction and organisational commitment. In this study, we examine how welfare technology reshapes care workers’ everyday care work and under what conditions those changes can be understood as upskilling, reskilling or deskilling.

**Objectives:**

To explore how welfare technology reconfigures working conditions and skill demands in home care and nursing homes.

**Methods:**

This qualitative study draws on eight vignette-based workshops with care workers (*n* = 45) from home care and nursing homes in four Swedish municipalities. The vignettes were used to prompt discussion about participants’ own experiences of everyday work with welfare technology. We analysed how care workers talked about welfare technology as supporting, changing or constraining their work, and interpreted these patterns through the concepts of upskilling, reskilling and deskilling.

**Results:**

Across the workshops, care workers described three pathways in which the use of welfare technology shaped their work. In one home care team, an integrated mobile system, coordinated alarm handling and app-based support for planning, documentation, medication sign-off and door access helped staff organise their work and maintain continuity in care. We read this as an example of upskilling. More often, however, care workers described fragmented documentation, parallel systems, frequent alarms and repeated interruptions. These conditions added administrative work, increased stress and reduced the time available for relational care, which we interpret as deskilling. Across both home care and nursing homes, care workers also described taking on new informal tasks such as troubleshooting devices, supporting older adults’ use of technology and helping colleagues manage digital systems. We see these added responsibilities as a form of reskilling, but when this extra work went unrecognised or unsupported, it often added pressure rather than skills development.

**Conclusions:**

Our findings show how care workers’ accounts of everyday work with welfare technology point to upskilling, reskilling and deskilling, depending on how welfare technologies are embedded in care work. For practice, our results point towards looking at what welfare technology requires from care workers in practice, not only whether different kinds of welfare technologies have been implemented.

**What is already known**

- Welfare technology is widely promoted in older adults’ care.

- Digitalisation can change care work in both supportive and burdensome ways.

- Little is known about the conditions under which welfare technologies support upskilling, reskilling or deskilling among care workers.

**What this paper adds**

- Upskilling among care workers was described in only one of the eight care teams in this study.

- Fragmented systems and alarm burden more often pushed work towards deskilling by interrupting care work and reducing time for relational care.

- Skill gains depended on integration, support and workable everyday routines.

## 1 Introduction

In recent years, welfare technology has become increasingly common in older adults’ care in Sweden and other Nordic welfare contexts (Frennert and Baudin, 2021; Kamp et al., 2019; Nilsen et al., 2016). Welfare technology is a Nordic concept (Österholm et al., 2025) used to describe technologies introduced in publicly funded municipal older adults’ care, including home care and nursing homes (Hämäläinen et al., 2026). In Sweden, welfare technologies such as mobile documentation systems, digital door access, alarms, sensors, monitoring cameras and social robots are introduced as part of efforts to improve care, respond to population ageing and address persistent difficulties in recruiting and retaining staff (Frennert and Baudin, 2021; Frennert et al., 2024; Svärdh et al., 2025, 2024). The Swedish National Board of Health and Welfare defines welfare technology as digital technology intended to maintain or increase safety, activity, participation or independence for people who have, or are at increased risk of having, a disability (Socialstyrelsen, 2023a). In policy and practice, welfare technology is therefore often presented as a way to support older adults’ independence while also making care work more efficient (Socialstyrelsen, 2023a, 2023b; Österholm et al., 2025).

At the same time, past research points to challenges and unintended consequences of digitalisation in care work (Lydahl and Hansen Löfstrand, 2020; Svärdh et al., 2024). In practice, welfare technologies are often introduced with limited involvement of care workers and without sufficient resources for care workers to learn how to use and master new technologies (Frennert, 2018, 2019). The implementation of welfare technologies also frequently depend on enthusiastic advocates, and when they leave, motivation to use the welfare technology and knowledge about how it should be used often diminishes or falters (Frennert, 2023). Previous research has also shown that welfare technologies reshape care workers’ roles and routines (Ertner, 2019; Persson et al., 2022; Svärdh et al., 2024). Svärdh et al. (2024), for example, show that welfare technology may affect agency in home care, as both care workers and care receivers may have limited influence over how welfare technologies are used in everyday care. Studies of the introduction of robotic cats in nursing homes illustrate that care workers are required to encourage and facilitate their use, which in turn places increased demands on care workers (Persson et al., 2022; Thunberg et al., 2020). Similarly, ethnographic research in Danish home care suggests that care workers must engage in affective and relational care practices to integrate welfare technologies into everyday care routines. Hence, care workers are expected to become ‘implementation agents’ who persuade colleagues and care receivers to trust and use the welfare technologies (Ertner, 2019, pp. 37–38). Furthermore, Bergschöld (2018) study of home care workers shows how a route-planning system may change the order, timing and coordination of visits, but also that home care workers may adapt or bypass the system when planned routes do not fit traffic or the practical realities of the workday. Gustafsson and Dannapfel (2025) highlight that leadership is needed to make welfare technology part of everyday work, while Rostad and Stokke (2021) show that the integration of welfare technology into long-term care depends on organisational conditions. Zander et al. (2023) similarly identify barriers and facilitators such as attitudes and competence. Thus, all aforementioned studies indicate that welfare technology has an impact of care workers. Hence, care workers may need to coordinate visits differently, encourage care receivers to use welfare technologies and adjust routines when welfare technology does not fit care situations. However, we found no studies that specifically illustrate how changes in roles and routines can be understood as upskilling, reskilling or deskilling among care workers. This raises a more specific question: when welfare technology is introduced into everyday care, does the use of welfare technology contribute to upskilling (i.e., expand care workers’ judgement and coordination), reskilling (i.e., shift care workers into new roles) or deskilling (i.e., erode relational continuity)? Prior research shows that professional development is important for job satisfaction and organisational commitment (Alshammari and Alenezi, 2023; Shiri et al., 2023). In an already pressured sector such as older adults’ care, opportunities for skill development may therefore matter both for retaining care workers and attracting people to care work (Jurij et al., 2023).

Against this background, this paper explores how welfare technology changes working conditions and skills in Swedish older adults’ care. We ask under what conditions the use of welfare technology contributes to upskilling, reskilling or deskilling among care workers. This paper contributes to research on welfare technologies by showing how the concepts of upskilling, reskilling and deskilling can be used to distinguish between different forms of skill change in everyday care work. Furthermore, our paper also illustrates that the boundary between reskilling and deskilling depends on how new welfare technology-related tasks are organised in practice. We use labour-process theory to examine how welfare technologies reshape task distribution, control and coordination in care work, and ethics of care to consider what these changes mean for attentiveness, responsiveness and relational continuity of care.

## 2 Theorising labour, skills and care in a digital transformation

In the context of welfare technology in older adults’ care, Braverman’s classical question of who controls technology, and to what ends, remains relevant (Braverman, 1998). Braverman criticised the Taylorist approach of factory and mass production for curtailing workers’ agency and professional judgement, rendering those who execute the tasks replaceable and easily supervised; while planning and control remain with management. More recent labour-process scholarship shows that control over work may also operate indirectly through targets and performance measures that shift accountability onto workers while reducing close supervision (Thompson, 2024). In relation to welfare technologies, prior research shows that they may centralise planning and redistribute accountability to care workers (Frennert et al., 2024). In some cases, welfare technology may introduce Taylorist features into care work by increasing standardisation, task segmentation and performance visibility (Frennert, 2018, 2019).

However, in contrast to industrial work, care work cannot be reduced to a set of standardised tasks. Relational care is itself skilled work (Himmelweit, 1999). The ethics of care framework foregrounds the affective, relational and situated dimensions of caregiving (Mol, 2008; Pols, 2012; Tronto, 2020, 2013). According to Tronto (2013), good care includes five linked elements: attentiveness, or noticing what the person needs; responsibility, or taking ownership of responding to the person’s needs; competence, or having and applying the skills to respond well; responsiveness, or attending to how the person is affected and adjusting the response accordingly; and caring with, the collective and political dimension of care that concerns how organisations and societies organise care in line with democratic values such as equality and trust. As such, good care is difficult to break down into standardised and time-measured tasks. In this paper, we use labour-process theory and ethics of care to analyse skill change by the use of welfare technology in care work. Labour-process theory helps us to analyse changes in task distribution, control and autonomy, while ethics of care helps us analyse what these changes mean for attentiveness, responsiveness and relational continuity.

### 2.1 The concepts: upskilling, reskilling and deskilling

To analyse how welfare technologies change skill demands, we use the concepts of upskilling, reskilling and deskilling. Upskilling is often framed as the process by which workers acquire new or more advanced competencies, often in response to technological change or shifting expectations in the organisation of work (Rafner et al., 2022). In older adults’ care, upskilling might involve developing digital literacy, mastering new software or adapting to remote monitoring systems (Kennedy and Yaldren, 2017). In this paper, we use upskilling to refer to working conditions where the use of welfare technology expands care workers’ competencies, control over work and professional judgement.

Reskilling is commonly understood as the acquisition of new skills due to changes in job tasks or occupational roles (Rafner et al., 2022). In care work, reskilling can be seen when digitalisation introduces new forms of service delivery, such as digital consultations, or requires workers to take on new tasks related to coordination, communication and technical troubleshooting (Pekkarinen et al., 2019; Sturt et al., 2020). In this paper, we use reskilling to refer to working conditions where the use of welfare technology shifts care workers into new coordinative and troubleshooting roles at the interface between people and welfare technology.

Deskilling, by contrast, refers to the erosion or displacement of previously valued skills (Rafner et al., 2022). Deskilling is often discussed in relation to Braverman’s critique of how capitalist production fragments and simplifies work in ways that reduce costs and increase managerial control (Martinaitis et al., 2021). In older adults’ care, deskilling may occur when relational and tacit care competencies are overshadowed by digital checklists, monitoring systems, or algorithm-driven planning tools (Vallor, 2015). In this paper, we use deskilling to refer to working conditions where the use of welfare technology backgrounds tacit and relational skills, such as attentiveness, responsibility, and responsiveness.

## 3 Methods

### 3.1 Study design

This study is part of a larger project aimed at understanding welfare technology in the Swedish older adults’ care sector. The project is designed as a mixed-methods programme, while this particular study is a qualitative, interpretive study based on vignette-based workshops with care workers (Burstin et al., 1980; Erfanian et al., 2020; Lee and Goh, 2020; Oltedal and Nygren, 2023). The workshops were used to explore how care workers recognised, interpreted and discussed everyday care situations involving welfare technology, and how the vignettes related to their own work practices.

### 3.2 Setting and participants

The study was conducted in four Swedish municipalities. The Nordic model of older adults’ care is characterised by universal access and is mainly financed through public funds and taxation (Rostgaard et al., 2022). National legislation defines rights and responsibilities, while the municipalities are responsible for assessing individual needs and organising the provision of care services (Szebehely and Meagher, 2018). Depending on older adults’ needs, care may be provided either at home or in nursing homes, within the same municipal organisation (Agerholm et al., 2024; Szebehely and Meagher, 2018). The overall aim is to enable older adults to maintain their independence and quality of life. Therefore, teams from both home care and nursing homes were included in the project.

In each municipality, we recruited one home care team and one nursing home team (eight teams in total). Participant recruitment was facilitated by municipal representatives who referred eligible teams. Participation was voluntary and all invited care workers received information about the study before providing consent. The care worker teams were selected because they had already provided background materials (photographs and descriptions of the welfare technologies they currently use, along with the rationale behind their use).

A total of 55 healthcare professionals agreed to participate, of whom 27 worked in home care and 28 worked in nursing homes. The sample consisted of 47 women and 8 men. Most participants were assistant nurses (*n* = 51) while four had care-related training. The majority (*n* = 33) had more than ten years of professional experience in older adults’ care, ten had 5–9 years of experience, and twelve had less than five years of experience. Seventeen participants, corresponding to almost a third of the sample, had a mother tongue other than Swedish; of these, seven worked in home care and ten in nursing homes. Due to scheduling conflicts, 45 healthcare professionals (7 men and 38 women) ultimately participated in the workshops. No managers were present.

### 3.3 Data collection: vignette-based workshops

Oltedal and Nygren's (2023) use of vignettes for reflections in practice and on practice is based on Schön's (Schon, 1959) concepts of 'reflection-in-action' and 'reflection-on-action.' Following Oltedal and Nygren (2023), we used vignettes as prompts for discussion. Vignettes are short, hypothetical scenarios that allow participants to respond to plausible and contextually relevant situations, making them a useful tool for exploring attitudes, values, and reasoning in relation to complex social practices (Barter and Renold, 2000; Burstin et al., 1980; Hughes, 1998).

**Fig. 1:**
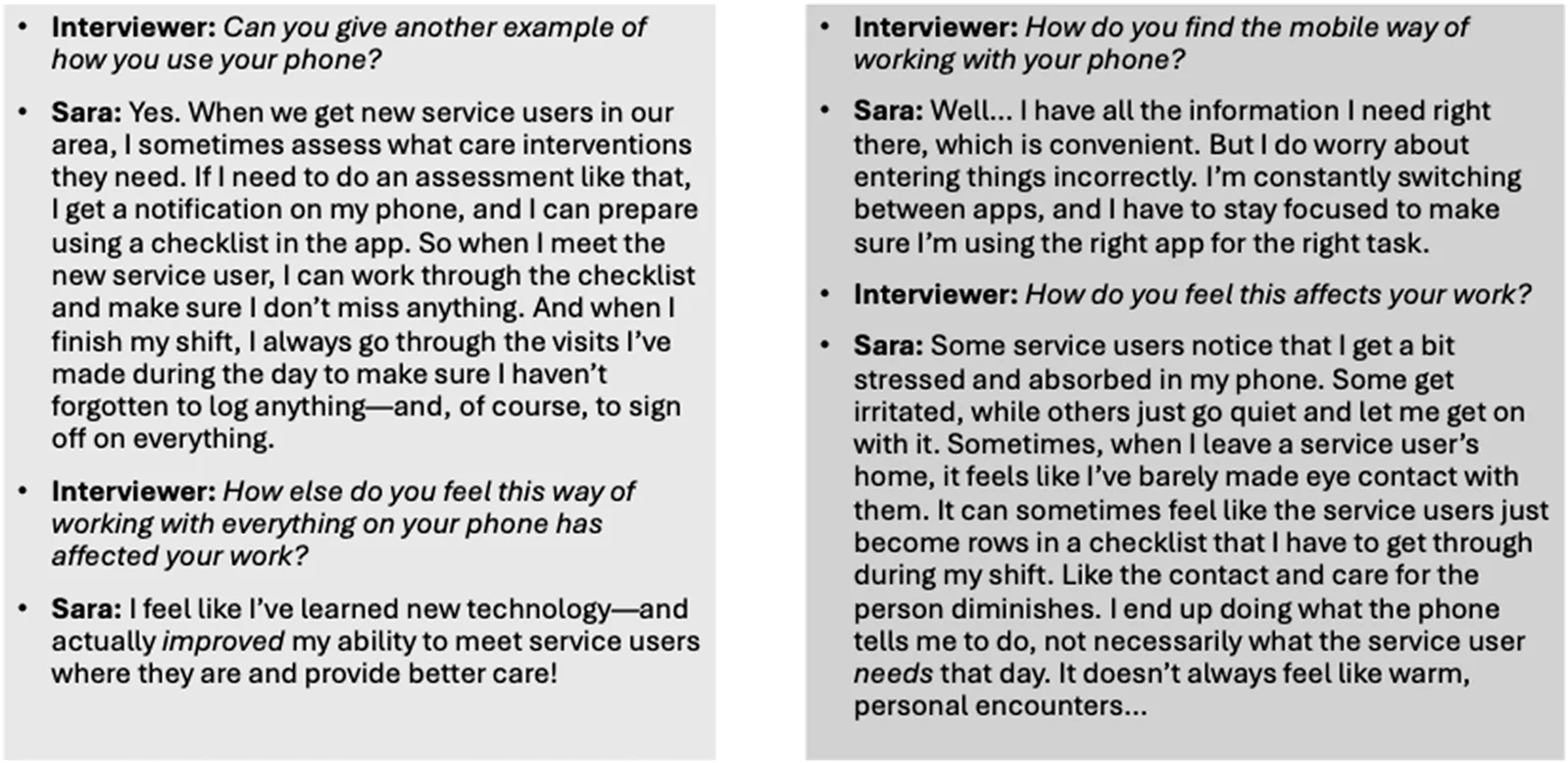
Extract from one of the contrasting vignettes regarding mobile ways of working.

The vignettes used in this study were informed by findings from two prior studies. In the first, we used photo-elicitation (Richard and Lahman, 2015) and asked care workers to photograph and describe the welfare technologies they currently use, along with the rationale behind their use. The second was a large survey study. Findings from these initial studies have been presented elsewhere (reference removed for anonymisation). The vignettes were developed around three overarching themes: (1) mobile ways of working (e.g. documentation apps, digital keys, medication apps); (2) welfare technologies for care receiver stimulation and engagement (e.g. robot pets, streaming services); and (3) welfare technologies for surveillance and monitoring (e.g. digital safety alarms, cameras, GPS-tracking watches). Each theme was paired with two contrasting vignettes to provoke reflection on everyday practice: one illustrating how the use of welfare technologies might lead to upskilling and another illustrating how it might contribute to deskilling (see Fig. 1).

Following Oltedal and Nygren (2023), we use the content of the vignettes to understand the care workers’ familiarity with the situations described in the vignettes (‘in practice reflections’) and the subsequent discussions to elicit care workers’ reflections on their own practice (‘on practice reflections’).

At each workshop, we used two brief, contrasted vignettes per theme, for a total of six vignettes at each workshop. The vignettes were accompanied by role-play in which the moderators presented the vignettes as interviews with an assistant nurse. To reduce cognitive and literacy demands often associated with reading prepared texts, the vignettes were presented orally by the moderators in this role-play format (Barter and Renold, 1999). After each pair of vignettes and role play, the moderator asked prompts such as: what in these vignettes aligns with your experience? What differs? Who benefits? Who carries additional workload? Which tasks move from whom to whom, or to the technology? Could you give a recent example? Where does this work well? Where does this not work so well? The vignette workshops were held at the care workers’ workplaces and lasted about two hours. All workshops were audio recorded and transcribed verbatim. The first author also took field notes and the second author served as the moderator. The field notes focused on interactions in the room, shared reactions to the vignettes, examples that generated particularly strong discussion, and contextual details that helped interpret the audio recordings. Data collection (a total of eight workshops) began in September 2024 and ended in February 2025.

### 3.4 Analysis

The analysis followed a multi-step process inspired by Braun and Clarke’s (2006; 2019) reflexive thematic analysis, combining inductive coding with theory-driven interpretation (Braun and Clarke, 2006, 2019).

The first stage involved both authors reading the transcribed workshop data and field notes. Both authors first carried out preliminary coding independently. Coding differences were discussed between the authors and resolved through comparison with the transcripts and field notes. We then developed a descriptive profile of each care team, drawing directly from the workshop discussions. For each team, we created structured tables detailing:

(1) which welfare technologies were available,
(2) how workers described their everyday use of these technologies, and
(3) how they emotionally and relationally experienced the integration of technologies into care routines.

The descriptive tables helped us to identify how welfare technologies were embedded in everyday work. We then conducted a comparative analysis across the teams. Notably, we observed that care workers’ experiences with welfare technologies were shaped more significantly by the type of care setting (home care versus nursing homes) than by municipal organisation. We therefore reorganised the descriptive tables into one for home care teams (Table 1) and one for nursing home teams (Table 2).

**Table 1:** Overview of digital setup and main sources of support and friction in the home care teams.

Team and participants	Main digital setup	Main sources of friction/support
Home care team 1 (3 women, 2 men)	Smartphone-based work with multiple apps for care planning, medication and tasks; staff safety alarm; desktop PC; paper binder in care receivers’ homes; private streaming services	Fragmented documentation across phone, desktop and paper; app instability; no digital door access; frequent alarm interruptions
Home care team 2 (3 women, 1 man)	Smartphone-based work with multiple apps, including door access; desktop PC; paper binder in care receivers’ homes; private streaming services	Fragmented documentation; rotating alarm responsibility; time spent troubleshooting care receivers’ private devices
Home care team 3 (6 women, 1 man)	Unified smartphone system for care plans, interventions, medication, scheduling, alarms and door access; two iPads for online grocery ordering; paper binder in care receivers’ homes; private streaming services	Integrated mobile workflow; reduced double work; app-based time adjustments; alarm triage supported continuity in care; added responsibility for online grocery support
Home care team 4 (4 women, 2 men)	Smartphone-based work with multiple apps, including door access; desktop PC; paper binder in care receivers’ homes; private streaming services	Fragmented documentation; digital door access only partly implemented; troubleshooting of care receivers’ private devices; dedicated alarm team reduced interruptions

**Table 2:** Overview of digital setup and main sources of support and friction in the nursing-home teams.

Team and participants	Main digital setup	Main sources of friction/support
Nursing-home team 1 (4 women)	Robot bird/cat; smart TV; private streaming via care workers smartphones; VR available but not in use; motion, door, camera and safety alarms; desktop PC	Fragmented documentation; paper still needed for medication details; technologies available but not in use; alarm fatigue; extra work with care receivers’ personal devices
Nursing-home team 2 (4 women)	Robot cat; smart TV; smartphones; medication app; iPads and activity tablets; private streaming via care workers smartphones; VR not in use; motion, door, camera and safety alarms; desktop PC	Multiple documentation systems; poor technical support; alarm fatigue; privacy concerns; unreliable GPS; no shared platform for knowledge exchange
Nursing-home team 3 (8 women, 1 man)	Robot dog/doll not functioning; smartphones; iPads mainly used for YouTube; motion and safety alarms; GPS watch; desktop PC	Fragmented documentation; paper signatures remained; alarm fatigue; broken or unused devices; extra work with care receivers’ personal devices
Nursing-home team 4 (6 women)	Robot cat used by one resident; smartphones; care receivers’ own TVs and smartphones; VR not in use; multisensory chair; motion and safety alarms; desktop PC	Alarm fatigue; limited knowledge-sharing; sporadic use of activation technologies; free apps had limited usefulness; extra work with care receivers’ personal devices

In the next, more theoretically driven stage, we applied the conceptual framework of upskilling, deskilling and reskilling to interpret how workers experienced digitalisation in terms of shifting skill demands. Drawing on Martinaitis et al. (2021) and Rafner et al. (2022), we coded both implicit and explicit references to welfare technology-related tasks, skill erosion, informal learning, cognitive overload and the reconfiguration of tasks and roles. We distinguished between comments directed mainly at the vignettes and comments in which the participants referred to their own work or recurring experiences. Participants’ accounts of situations where welfare technology gave them better overview and control over work, or room for professional judgement and relational care were coded as upskilling. Accounts of welfare technology-related tasks such as coordination, troubleshooting and supporting care receivers with technology were coded as reskilling when the welfare technology-related task were described as part of the work and supported in practice. Accounts of situations where welfare technology created interruptions, fragmented work, led to double documentation or reduced time for relational care were coded as deskilling. The same task could therefore be coded differently depending on how the circumstances were described. Troubleshooting, for example, was coded as reskilling when it was supported by the care organisation and part of the care workers’ role, but as moving towards deskilling when troubleshooting was added to care work without organisational support and time in the planning schedule. Care receivers’ own technologies such as smartphones, tablets and other devices were first kept analytically separate from municipal welfare technologies. However, during the analysis, we noticed that helping care receivers with their own technologies was frequently mentioned by the care workers as part of their everyday care work. We therefore included the care receivers’ private technologies, because participants described them as affecting working conditions and skill demands in home care and in nursing homes (Table 3).

**Table 3:** Examples of how participants’ accounts were interpreted as upskilling, reskilling and deskilling.

Quotations	Initial codes	Subthemes	Concept
“all information, everything, you can bring up in the phone”	Access to information	Overview	Upskilling
“we use iPads, for example when we do food orders, we do it together with the care receivers’ so that they can see what to order and different choices”	Digital food ordering with care receivers	New tasks/routines	Reskilling
“we spend an enormous amount of time on it [the care receivers’ private technologies] instead of doing what we are supposed to do … sometimes they [the care receivers] have pressed all the buttons”	Troubleshooting; added responsibility; not doing what they are expected to do	Informal technical support	Reskilling with risk of deskilling
“we have all these different systems and apps…different routes to get the information we need before a visit”	Several systems; several applications; scattered information	Fragmentation	Deskilling

### 3.5 Ethical considerations

This study was conducted in accordance with the ethical principles set out in the Declaration of Helsinki. All participants provided informed consent, and the study protocol was reviewed and approved by the Swedish Ethical Review Authority, diary number 2023–05,238-01. The rights, safety and wellbeing of participants were prioritised throughout the research process. Throughout the presentation of the findings all identifying details are anonymised. We use pseudonyms and include anonymised verbatim quotations.

## 4 Results

**Fig. 2:**
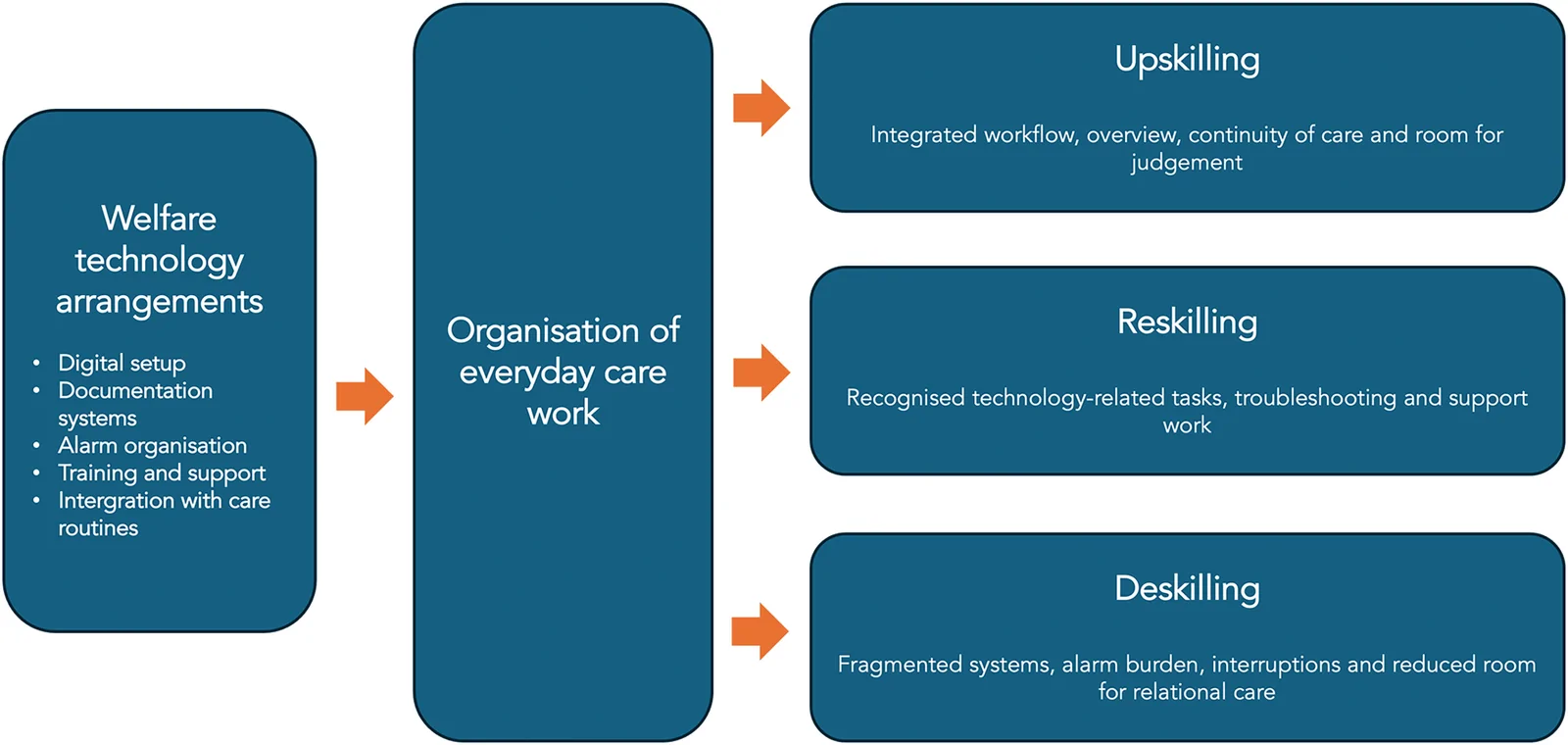
Upskilling, reskilling and deskilling pathways shaped by the use of welfare technology.

To address our research question - how do welfare technologies reconfigure working conditions and skill demands in terms of upskilling, reskilling and deskilling - the findings are organised through the analytic concepts of upskilling, reskilling and deskilling. Each concept includes subthemes that show how welfare technologies changed everyday care work and under what working conditions care workers’ skills were supported, reshaped or made harder to use (Fig. 2).

### 4.1 Upskilling: overview, coordination and room to act

In our analysis, the clearest example of upskilling was found in one home care team. Home care team 3 differed from the other care teams because welfare technologies were integrated into everyday care routines.

#### 4.1.1 Overview and access to information

Care workers in home care team 3 used smartphone applications for documentation, door access, medication administration and monitoring of care tasks. They had adopted a mobile way of working. As one worker put it: *“Now that we have everything on the phone, it saves time and avoids double work, that is working both on the computer and on the phone or with paper”.* (Isak, assistant nurse, home care team 3) Another care worker described the phone as giving them direct access to information during care situations: *“All information, everything, you can bring up in the app”.* (Kerstin, assistant nurse, home care team 3)

The team conveyed a sense of having continuous access to information, which they associated with improved communication and responsiveness to care receivers. They described that when care workers documented directly after a care visit, the next care worker could use this information at the next visit to the care receiver. As such, the use of smartphones and the mobile workflow were described as supporting care continuity across care visits.

#### 4.1.2 Room to act in daily care

Home care team 3 also explained how the mobile documentation could be used to make care needs visible. One care worker gave an example of a morning visit to a care receiver that had been allocated 30 min but in practice took 50 min instead. She explained that she would write this into the care plan and ask for more time from the needs assessor: *“they [needs assessors] read the care plan and can see in detail what we do and how much time it actually takes. They then change the times for the visits so that we get enough time for each care receiver”.* (Erica, assistant nurse, home care team 3)

During the workshop with home care team 3, the organisation of alarms was also mentioned. All alarms were filtered through a call centre, which assessed urgency before contacting the care worker closest to the care receiver. This arrangement was described as supporting relational continuity, especially when working with people with dementia. As one worker explained: *“We work with people with dementia. They get very stressed and upset if someone they don’t know comes to their home in an already stressful situation”.* (Stina, assistant nurse, home care team 3).

In home care team 3, upskilling was linked to a work organisation in which welfare technologies were integrated into daily care routines and supported care workers’ overview of work, coordination of care and room to act. Mobile-first documentation, in-app time adjustments and alarm triage through a call centre were described as reducing double work and supporting more flexible and continuous care. We interpreted this as the clearest example of upskilling in our material.

### 4.2 Reskilling as new welfare technology-related care work

Across the workshops, care workers described taking on new tasks related to welfare technology and care receivers’ own devices. New tasks included troubleshooting, adapting digital devices to care situations and using available welfare- and private technologies to support social contact and emotional connection. In our analysis, these practices were interpreted as reskilling because the new tasks changed what the care workers needed to know and do in everyday care work.

#### 4.2.1 Relational uses of welfare and private technology

Across several workshops, care workers described using private phones and streaming accounts to play music for care receivers. One participant noted*, “We just use our phones to play music for the care receivers, but the ads are annoying, they can upset care receivers”* (Katarina, assistant nurse, home care team 2), referring to the limitations of the free version of Spotify. In another home care team, music was described as part of knowing the care receiver: *“I use music a lot. I have played both Rolling Stones and ABBA today. Music often makes it easier to handle them [care receivers], because they become happier”*. (Lisbeth, assistant nurse, home care team 4)

In nursing homes, music apps were used to create a calmer atmosphere in shared spaces. Smart TVs were also used relationally: *“You put on a programme and sit with the care receivers. You see if the care receivers sing along and if they like the programme. If they don’t, you try something else”.* (Alice, assistant nurse, nursing home team 3) Other care workers described playing music or using smart TVs together with the care receivers as a way of creating contact: *“we get closer contact with them [care receivers] if we sit with them and watch the same programme. We can talk about what happens, which is sometimes easier than making up a topic to talk about as we are different generations”*. (Robin, assistant nurse, nursing home team 1) Similarly, another care worker explained: “*You get a connection and have something to talk about”*. (Alex, assistant nurse, nursing home team 2)

Care workers also described taking on new digital routines together with care receivers. In one home care team, iPads were used for online food ordering. One care worker explained: *“we use iPads, for example when we do food orders, we do it together with the care receivers’ so that they can see what to order and different choices”.* (Anita, assistant nurse, home care team 3) Online food ordering was described as making it possible for care receivers to participate in food shopping, as well as reducing the physical strain for care workers. Earlier, care workers had gone food shopping in local stores for the care receivers. When asked what happened with the time saved for not having to go to the local stores, one care worker answered: *“We use it with the care receivers”.* (Ulla, assistant nurse, home care team 3)

Welfare technologies specifically introduced for activation purposes were also discussed in the workshops. For example, in one workshop, a robotic bird that malfunctioned was described: *“Something happened to the bird. Suddenly it just was quivering and did not stop. A care receiver got stressed and ‘killed’ it by putting the robotic bird into his coffee”.* (Ellen, assistant nurse, nursing home team 1). A similar story was shared about a robotic cat: “*Initially the care receivers were fascinated by the robotic cat but after a while they got upset and said that it did not behave like a real cat”.* (Ylva, assistant nurse, nursing home team 4). These narratives were described by the participants as part of ongoing relational experimentation between care workers and care receivers.

Thus, the participants described how welfare technology as well as private technologies had become part of situated and relational care. The care workers had to learn when, for whom and how welfare technologies and private technologies could be used to calm the care receivers, as well as when the technologies could be used to create contact or provide a topic for conversations.

#### 4.2.2 Informal technical support

In several municipalities, care workers were also regularly asked to assist with digital troubleshooting for care receivers’ personal devices, such as adjusting smartphone settings, fixing a Chromecast or explaining how to use a TV. One care worker described how much time informal technical support could take: *“we spend an enormous amount of time on it [the care receivers’ private technologies] instead of doing what we are supposed to do … sometimes they [the care receivers] have pressed all the buttons”.* (Hedvig, assistant nurse, home care team 4) Similarly, another care worker described handling TVs as a recurring task: *“Troublesome remote controls and TV sets are what we spend time on”.* (Liv, assistant nurse, nursing home team 4)

In one workshop, a care worker mentioned a male colleague as *“the one everyone turned to for technical help”.* (Elisabeth, assistant nurse, nursing home team 4) His informal role extended beyond traditional care support as he was also the one sent to care receivers who needed technical support. In another workshop, a care worker described calling a younger colleague when the care receivers’ private technologies did not work: *“I just can’t handle it. I call a younger colleague. It is hopeless”.* (Lykke, assistant nurse, nursing home team 3)

Reskilling was uneven. In many teams, a few workers became the unofficial ‘technology experts’, while others relied on their more tech-savvy colleagues. Sometimes the use of welfare technologies and private technologies became part of the care worker’s role and changed what care workers needed to know and do. At other times, handling welfare- and private technologies was added on top of ordinary care work without time or support. In the latter cases, we interpret this as reskilling with a risk of moving towards deskilling.

### 4.3 Deskilling through alarms, fragmentation and fragile infrastructure

Across the workshops, care workers often described the use of welfare technologies in ways that fragmented their work. In our analysis, deskilling did not mean a loss of ability. Rather, it referred to shifts in how care tasks were carried out, how knowledge was accessed, and how digital infrastructures intersected with care activities. Participants described working conditions that was increasingly organised around interruptions, alerts, and documentation demands that made it harder to remain present with care receivers.

#### 4.3.1 Alarms and interruptions

One of the most frequently mentioned welfare technologies contributing to deskilling was digital alarm systems. During the workshops, care workers described the sound of frequent alerts: “*I get quite stressed when it keeps pinging while I am with someone”. (Sophia, assistant nurse, nursing home team 3)* The alarms were not only disruptive for care workers but also for care receivers. As one of the care workers said: *“Some care receivers comment on it too, like; Now you have to go, I should stop talking now”.* (Elsa, assistant nurse, nursing home team 3).

In another nursing home, care workers described the constant sound of alarms more bluntly: *“The alarms go off all the time. All the time. It is never calm and silent”.* (Charlie, assistant nurse, nursing home team 4) One participant added: *“You are really tired after a work shift”*. (Kim, assistant nurse, nursing home 4) Another care worker described the effects of the alarms as mental tiredness: *“You get tired in the brain, I would say”*. (Lo, assistant nurse, nursing home 4)

In one home care team, carrying the alarm phone was described as a source of constant disruption. As one participant noted*: “You are always interrupted and it means you do not finish anything”.* (Britta, assistant nurse, home care team 2). Similarly, a care worker explained: “*When you get an alarm, you have to let go of what you are doing to respond to the alarm”.* (Bo, assistant nurse, home care team 2) When asked whether the use of digital alarms such as sensors and cameras freed time for them, one participant answered: *“No. Absolutely not”.* (Mika, assistant nurse, home care team 4)

Several care workers described the difficulty of interpreting alert severity, whether an alarm signalled urgency or less urgent matters. *“You always think, maybe this is the one alarm you should not ignore”.* (Calle, assistant nurse, home care team 1) Another added, “*You lose the thread”.* (Lina, assistant nurse, home care team 1) referring to the feeling of being pulled out of a care moment or task. Our analysis revealed that the organisation of alarms shaped the outcome. When alarms were filtered or handled by a special alarm team, care workers described fewer interruptions. When the alarms went directly to care workers, the frequent alarms interrupted ongoing care and made it harder to stay with care receivers.

#### 4.3.2 Fragmented documentation and scattered information

In several workshops, care workers described how they skimmed documentation or prioritised only certain colleagues’ notes: *“I only read certain colleagues’ notes because some colleagues just write too many uninformative things”.* (Fatima, assistant nurse, nursing home team 4). The care workers’ choices were not framed as dismissive but as pragmatic responses to information overload and system limitations.

Participants also described moving across multiple platforms and multiple national registers, alongside binders, whiteboards and oral exchanges. Rather than streamlining work, digitalisation was described as scattering information, making it hard to trace what had been done or what needed to be known. One participant summarised this experience: “*You just don’t know where to look sometimes”.* (Kerstin, assistant nurse, nursing home team 2) In one nursing homes, a care worker described the number of systems in use:” Right *now we use many checklists and notes. We write daily notes on a stationary computer, and we have several systems such as Senior Alert, BPSD and Treserva. But none of the systems are user friendly”.* (Rita, assistant nurse, nursing home 1) In another nursing home team, participants described the many information routes: *“we have all these different systems and apps…different routes to get the information we need before a visit”*. (Yonna, assistant nurse, nursing home team 4).

In home care, the same problem with fragmented and scattered information appeared when the phone applications did not have access to all information. One participant explained that the medication app only showed the time for medication, but not the full medication list: *“The app on the phone tells you when the care receivers’ should have their medication. To know what kind of medication each one of them should have, you have to check a physical list”.* (Helen, assistant nurse, home care team 4) Her colleague added: *“In the phone it only says: give the dose at eight o’clock. It does not say how many tablets”.* (Cecilia, assistant nurse, home care team 4)

Thus, as seen in the cases above, welfare technology did not replace old routines. Instead, care workers had to combine several sources in order to know what to provide care.

#### 4.3.3 Fragile infrastructure and lack of support

A third working condition that we interpreted as leading to deskilling concerned infrastructure and support. Care workers described systems that lagged, Wi-Fi that did not work and welfare technologies introduced before the local conditions were ready. One care worker described this as: *“Sometimes we have to run around and chase a Wi-Fi connection”.* (Rut, assistant nurse, nursing home team 3) Her colleague further explained: *“It takes a long time to start the applications. It just lags, lags and lags”.* (Annette, nursing assistant, nursing team 3).

In workshop after workshop, care workers said they were expected to start using new welfare technologies without formal training. An often-heard phrase across both nursing homes and home care services was some version of: *“we just figured it out.”* At times, testing led to moments of surprise and discovery; at others, it resulted in confusion. In one of the workshops, for example, care workers explained that when new colleagues started, *“they learn during job shadowing. They do not get any training in how to use different kinds of welfare technologies. They just follow us and ask questions when needed”.* (Mary, assistant nurse, home care team 4).

Across all four municipalities and in both home care and nursing home settings, similar patterns were observed. Care workers spent time making welfare technologies work, finding information and compensating for systems that did not fit daily care work. Under these conditions, the pathway to deskilling emerged.

## 5 Discussion

Our study contributes to debates on welfare technology, care work and skill change (Hämäläinen et al., 2026; Naseer and Dellve, 2025) by examining how welfare technologies become part of the organisation of everyday care work. Although we identified three pathways of skill change, the findings point most strongly towards deskilling. Most care teams described fragmented systems, interruptions due to alarms, and unsupported troubleshooting (i.e., deskilling), while only one care team described a configuration in which welfare technologies supported upskilling. Reskilling appeared across both home care and nursing homes. New welfare technology-related tasks became skills development when recognised and supported but slide towards deskilling when added informally without organisational support and resources in terms of extra time.

By connecting research on welfare technology with labour-process theory and ethics of care, our analysis illustrates how welfare technologies reorganise care tasks and shape the conditions under which skilled and responsive care can be practiced. Previous research shows that welfare technologies must be adapted and domesticated in local care practices (Frennert, 2018; Frennert et al., 2024; Glomsås et al., 2020; Svärdh et al., 2025). Our study extends previous research by showing how care workers account on of the adoption and domestication of welfare technologies can be interpreted as upskilling, reskilling and deskilling.

In the most supportive configuration, welfare technology was embedded in a coherent digital workflow. In one home care team, smartphones were used to gather all documentation, enable digital medication sign-off, open doors, and access information about care receivers through one connected system and workflow. Care workers documented the care receivers’ changing needs, and needs assessors used this documentation to adjust the time slots allocated to each care receiver. Our upskilling case presents a different dynamic from Braverman’s (1998) classical deskilling thesis, in which planning of work tasks are separated from their execution. Our analysis shows that the digital workflow, in one of the home care teams, partly reconnected planning and execution of care tasks. Thus, in this case, care workers’ documentation influenced time allocation. As such, the digital workflow connected care workers’ judgement about the care receivers’ needs to organisational decision-making. In labour-process terms, the digital workflow appeared to increase care workers’ control over aspects of care by connecting situated knowledge to planning and time allocation (Braverman, 1998; Thompson, 2024). Hence, this particular case represents the most observable pathway to upskilling in our material.

Most workshop participants, however, described less coherent welfare technology arrangements. Care workers often needed to move between phones, paper lists, stationary computers and binders, while also responding to different alarms and providing technical support to care receivers and colleagues. The dominant deskilling pathway shows a more constraining configuration of welfare technology usage. Thus, care workers remained responsible for delivering good care, but they had to do so through welfare technologies that did not sufficiently support their daily work and through fragmented infrastructures. The deskilling pathway extends labour-process accounts by identifying a technology-specific mechanism in older adult’s care (Baines et al., 2024; Braverman, 1998; Thompson, 2024). Classical accounts of deskilling emphasise task simplification and managerial control (Braverman, 1998). Furthermore, Baines et al. (2024), for example, show how standardised care can constrain professional judgement even when workers retain their professional commitments and knowledge. Our study points to a related but more technology-specific mechanism: fragmented welfare technologies, alarm burden and unsupported troubleshooting redistributed responsibility to care workers without giving them the time and overview needed to exercise relational care. Alarms, different apps and lagging system made it harder for them to provide good care in the sense of Tronto’s concepts of attentiveness, responsibility and responsiveness (Tronto, 2020). Care workers had to divide their attention between the care receivers and the demands of the welfare technologies. Thus, the working conditions weakened the possibilities for relational care. As such, attentiveness to care receivers was shaped by the organisation of welfare technologies. Hence, deskilling in older adult’s care occurred in our material mainly through fragmentation and interruption rather than task simplification or replacement. Our findings resonate with Lydahl’s (2024) point that welfare technologies may enact some values, such as safety and availability, while at the same time undermining others, such as calmness, continuity of care and responsiveness. Our study indicates that deskilling in care work involves reduced control over the working conditions needed to provide good care.

Reskilling, in our material, appeared in the new technology-related tasks that care workers took on. Technology-related tasks broadened care work when they were recognised and supported. When they remained informal, they redistributed responsibility to care workers without increasing their control over the work process. The reskilling pathway appeared to be conditional: new technology-related tasks could support skill development when recognised and resourced but move towards deskilling when they relied on care workers’ goodwill without corresponding organisational support. Such mixed trajectories support the argument that upskilling and deskilling can co-exist within the same role (Martinaitis et al., 2021). Our analysis of the reskilling pathway refines the understanding of skill change in older adults’ care by showing that reskilling is not simply a matter of learning new welfare technology-related tasks. Rather, reskilling depends on whether new welfare technology-related tasks are recognised as part of care work and supported by the care organisation through time, training and organisational responsibility.

Our study adds to the literature on welfare technology implementation (Bergschöld, 2018; Borg et al., 2023; Lydahl and Davidsson, 2024; Svärdh et al., 2025). Previous research has shown that information, managerial facilitation and user involvement shape whether welfare technologies are accepted and used in practice (Glomsås et al., 2020). Our findings make visible that the use of similar welfare technologies can lead to different skill outcomes across different care settings (see Table 4). Thus, similar welfare technologies may support relational care in one setting and fragment the continuity of care in a different care setting.

**Table 4:** Empirical examples of welfare technology arrangements and skill-change pathways.

Welfare technology application	Upskilling pathway	Reskilling pathway	Deskilling pathway
**Smartphone-based care management** (e.g., digital applications for care planning, medication management, scheduling, task coordination, secure door access)	Integrated mobile workflow supported access to information, continuity across visits and documentation that could influence time allocation.	Care workers learned new routines for documentation, coordination and task management.	Partly integrated systems required movement between phone, desktop and paper, leading to fragmented records and duplicate work.
**Remote monitoring** (e.g. alarms and alerts from surveillance cameras, environmental sensors and personal security alarms)	Alarm triage reduced interruptions and supported continuity of care.	Care workers developed skills in interpreting alerts and coordinating responses.	Direct and frequent alarms interrupted care, increased stress and reduced attention to relational care.
**Streaming services, companion robots and virtual reality** (e.g. for care receivers’ comfort and engagement	When matched to the care receiver and situation, welfare technologies could support contact, calm and shared activity.	Care workers learned to select and facilitate welfare technology use in relation to the care receivers’ responses.	Malfunctioning, underused or unclear welfare technologies added work and could displace relational care.
**Handling care receivers’ private technologies**	Helping with private devices could support calmer interactions and digital confidence.	Care workers took on informal troubleshooting and guidance alongside ordinary care tasks.	Unsupported troubleshooting took time from planned care and left care workers responsible for problems they could not always solve.

### 5.1 Implications for policy and practice

For practitioners and policymakers, Table 4 highlights that municipalities and care organisations should evaluate welfare technology not as standalone technical solutions, but as part of wider care configurations. Evaluations of welfare technology should therefore focus on how welfare technologies reorganise work, responsibility and care quality.

Our findings have several implications for policy and management:

First, care organisations and municipalities need to make continuity and fragmentation visible, such as how many systems care workers need to use and the extent to which work is duplicated across several systems.

Second, alarm burden should be treated as an organisational outcome rather than an individual problem for care workers to manage. Care organisations and municipalities need to examine, for example, how many alarms are generated, who receives and responds to the alarms, and what kinds of care are interrupted when alarms redirect attention from one care receiver to another.

Third, troubleshooting technologies, helping care receivers with their devices and supporting colleagues in their use of welfare technologies are not peripheral tasks. Our study shows that technology-related work is part of contemporary care work and should be made visible and resourced.

Fourth, the use of welfare technology reshapes the labour process of care. As such, it should not be individual care worker’s responsibility to make welfare technologies work in everyday practice. Instead, the workings of welfare technologies should be treated as an organisational concern and responsibility.

### 5.2 Limitations

This study has several limitations. First, it does not capture older adults’ own voices directly. Instead, their perspectives appear indirectly through care workers’ accounts of how care receivers responded to welfare technologies. Second, the study is based on vignette-based workshops in four municipalities. The findings therefore need to be understood in relation to these settings and to the organisation of Swedish municipal care. The inclusion of both home care and nursing homes made it possible to compare how welfare technologies were configured across central areas of Swedish older adults’ care. However, home care and nursing homes differ in work organisation, spatial conditions, and alarm routines. Some findings are therefore likely to be care-setting specific. Third, the selection of teams was facilitated by municipal representatives, which may have influenced the findings. However, the findings do not indicate an overly positive assessment of welfare technology in relation to improving work-related skills. Forth, the vignettes were deliberately constructed to contrast situations that could be interpreted as upskilling or deskilling. It may have orientated participants towards these categories during the discussions. To reduce the risk, we treated the vignettes as prompts and our analysis focused on participants’ own examples and recurring reflections on everyday work. Fifth, the boundary between welfare technologies and care receivers’ private devices was difficult to maintain analytically. Although welfare technologies and care receivers’ private devices were initially treated as distinct, it became apparent during the workshops that the boundaries between welfare technologies (paid by the municipalities) and the care receivers’ private technologies were blurred in everyday care work. Care workers were expected by care receivers to help with remote controls, TV sets, and smartphones, which in turn affected their working conditions. As such, our study describes the wider ecology of technologies encountered in care work rather than only municipally provided welfare technology. Thus, the findings indicate that both welfare technologies and care receivers’ private technologies are important features of contemporary care work.

## 6 Conclusion

In this paper, we have shown how welfare technology becomes part of the labour process of care. Welfare technologies enter everyday care work through documentation systems, alarms, digital keys and medication apps, and in doing so they reorganise who coordinates care, who carries responsibility for care and how much room care workers have to act.

Upskilling, reskilling and deskilling are different ways in which welfare technology organises care work. Welfare technology supported upskilling when it gave care workers a better overview of daily care work and strengthened their room to act. Reskilling appeared when new welfare technology-related tasks were recognised as part of care work. However, reskilling could shift towards deskilling when fragmented systems, repeated alarms and unsupported troubleshooting reduced care workers’ capacity for relational care.

Attentiveness, responsiveness and relational continuity of care depend on time, information and room to act. The use of welfare technology shapes what care workers can prioritise by directing their attention and creating new tasks. As such, the use of welfare technology matters for the ethics of care, as welfare technologies are part of the conditions under which good care can and cannot be provided. Thus, without strong welfare technology integration into care work and ongoing support, municipalities risk paying twice: once for the welfare technology and again in the hidden costs of fragmentation, alarm fatigue and foregone care.

## Declaration of generative AI use

The authors used ChatGPT for language editing (grammar and spelling). No generative AI was used to create scientific content, interpret findings or draw conclusions.

## Funding

This work was co-funded by the Swedish Research Council for Health, Working Life and Welfare (grant number 2023-00174). Open access funding was provided by 10.13039/501100014149Lund University. The views expressed are solely those of the authors and do not necessarily reflect those of the funding body, which cannot be held responsible for them.

## CRediT authorship contribution statement

**Susanne Frennert:** Writing – review & editing, Writing – original draft, Validation, Methodology, Investigation, Formal analysis, Data curation, Conceptualization. **Katrin Skagert:** Writing – review & editing, Methodology, Investigation, Formal analysis, Conceptualization.

## Declaration of competing interest

The authors declare no conflict of interest. The funders had no role in the design of the study; in the collection, analyses, or interpretation of data; in the writing of the manuscript, or in the decision to publish the results.

## Data Availability

To protect participants’ privacy, the full dataset is not publicly available. If the Swedish Ethical Review Authority permits, the corresponding author can share it in Swedish on reasonable request.
