# Supplementary material for: Upskilling, reskilling and deskilling when using welfare technologies in Swedish older adults’ care: A vignette-based qualitative study across four municipalities
Source: Int J Nurs Stud Adv. 2026 Aug 20;11:100665. doi: 10.1016/j.ijnsa.2026.100665 (PMC13583603; doi:10.1016/j.ijnsa.2026.100665)
Supplement: Supplementary file 1 [file mmc1.pdf]

Appendix 1.

Up-skilling vignette (Home care): Mobile ways of working (e.g. documentation apps, digital keys, medication apps)

Interviewer: *Sara, can you tell me how you use your phone during a work shift?*

Sara: Yes… I always start my shift by logging into my smartphone. It has everything I need for my shift—my tasks and all the service user information.

Like yesterday, for example. My first visit in the morning was to Stina, who has chronic arthritis. Before I even got there, I could see in the app that she'd had a lot of pain during the night. So when I arrived, I knew right away that I should give her pain relief, and then Stina and I could start the day together once she'd had it. We could talk about what she needed next, and so on. And of course, I signed off on the medication in the app right away.

It was a bit like that when I went to see Martin, who has dementia. He's often a bit anxious and confused when you arrive. A colleague had left a note about Martin in the app, describing how he was yesterday. So, on top of seeing what he needs help with at a specific time, the note helped me adjust my approach to how Martin was feeling *that* day. It made it much easier for Martin and me to connect and work together at his home. Afterward, I logged my visit in the app and, if needed, left a note for the colleague coming after me.

We do this a lot—writing tips for each other about what to keep in mind with different service users. It's really important for handing over to the next colleague or the night shift in a good way.

Interviewer: *Can you give another example of how you use your phone?*

Sara: Yes. When we get new service users in our area, I sometimes assess what care interventions they need. If I need to do an assessment like that, I get a notification on my phone, and I can prepare using a checklist in the app. So when I meet the new service user, I can work through the checklist and make sure I don't miss anything. And when I finish my shift, I always go through the visits I've made during the day to make sure I haven't forgotten to log anything—and, of course, to sign off on everything.

Interviewer: *How else do you feel this way of working with everything on your phone has affected your work?*

Sara: I feel like I've learned new technology—and actually *improved* my ability to meet service users where they are and provide better care!

De-skilling Vignette (Home care): Mobile ways of working (e.g. documentation apps, digital keys, medication apps)

Interviewer: *Sara, can you tell me how you use your phone during a work shift?*

Sara: Yes… I always start my shift by logging into my smartphone. We have a special app to unlock doors when we visit our care recieivers—no keys needed. Then there's another app where I log and sign off on everything I do with a service user. Oh, and we also have a separate app for signing off on medications. I have to open that one every time I've given someone their medicine.

Interviewer: *Okay. And at the end of your shift, you just log out, close the apps, and you're done? Or…?*

Sara: No, not quite. After I've finished my visits, I have to end my shift at the office. I log into one of our desktop computers there and update all the service users' records. Then I'm done.

Interviewer: *How do you find the mobile way of working with your phone?*

Sara: Well… I have all the information I need right there, which is convenient. But I do worry about entering things incorrectly. I'm constantly switching between apps, and I have to stay focused to make sure I'm using the right app for the right task.

I forgot to mention earlier, but when I'm giving medication, for example, I'm standing there with two apps open *at the same time*—and the service user right in front of me. In one app, I verify the correct dosage, and in the other, I sign off that I've given the medication. If the service user starts talking to me at the same time, I easily lose track. It's happened before…

Interviewer: *How do you feel this affects your work?*

Sara: Some service users notice that I get a bit stressed and absorbed in my phone. Some get irritated, while others just go quiet and let me get on with it. So, yeah—it's a lot of nose-in-phone time. Sometimes, when I leave a service user's home, it feels like I've barely made eye contact with them.

It can sometimes feel like the service users just become rows in a checklist that I have to get through during my shift. Like the contact and care for the person diminishes. I end up doing what the phone tells me to do, not necessarily what the service user *needs* that day. It doesn't always feel like warm, personal encounters…

De-skilling Vignette (Home Care): Welfare technologies for care receiver stimulation and engagement (e.g. robot pets, streaming services)

Interviewer: *Anna, can you tell me about your day? Did you use any kind of technology with your service users today?*

Anna: Yes, I suppose so. We've been given a robotic cat to use with our care receivers. But I don't know—it feels a bit odd. Like with Karin today. It's like putting a toy in her lap. She did smile a little when the cat started purring, but I didn't have time to talk much. I had to clean up and log the documentation on my phone. The tech often malfunctions, too. Today, the cat just stopped working suddenly, and Karin thought it had "died." I had to quickly fix the batteries, but by then, she was already upset. Not exactly a success.

But I guess it *can* free up time for other tasks if the cat keeps the care receiver's calm.

Since the pandemic, we've also been helping our care receivers to stay in touch with family through video calls. But the tech often glitches, and I end up being more of an IT technician than a caregiver. That doesn't feel great. And then I feel a bit redundant—like today, when I helped Britta connect with her daughter. She'd rather talk to *her* than to me.

Interviewer: *Is there anything else you do to stimulate your service users?*

Anna: Stimulate... Well, I might put on music they recognize and like. I bring my tablet if they don't have their own. I did that with Karin yesterday, actually. But I'm not sure it's *stimulating*... More like something playing in the background while I do other things.

Some service users have smart TVs, and I'll help them find shows they like. But then they just sit and watch TV, and we don't have many meaningful conversations. Though it *does* mean I can get more done—less time spent talking, more time for tasks.

Interviewer: *Is there anything else you've noticed about how this affects your work?*

Anna: Hmm, maybe. I feel like I'm becoming more of a technical support service. Less about working *with* the people we're there for. So it's more about emptying the dishwasher, cleaning, measuring out medications... More routine work, less actual care.

Up-skilling Vignette (Home Care): Welfare technologies for care receiver stimulation and engagement (e.g. robot pets, streaming services)

Interviewer: *Tell me about your day, Anna!*

Anna: Well, today I brought the robotic cat Mimmi along on my rounds. The first care receiver I visited was Lars, who has dementia. When he held Mimmi in his lap and she started purring, he lit up—just like he always does—and talked about his old pets, stroking her. He relaxes with the cat, and it makes things like shaving him or getting him to take his medication much easier.

Interviewer: *Is there anything else you've noticed about using the cat?*

Anna: Oh, it helped me get to know Lars better. That means I can provide the care he *really* needs. And I think the cat—and our conversations about memories—do a lot for him, too. It gives him a good start to the day.

Interviewer: *Who did you visit after Lars?*

Anna: Greta. She hadn't had a good morning—poor sleep, pain, all that. But when she held Mimmi, she could focus on the cat and talk about *her* instead of getting upset when I helped her. That let me support her better. She misses her family, who live quite far away, so we decided to try video calling her son and grandchildren later that afternoon when I came back. It felt good to ease her loneliness a bit.

I actually do the same for Bengt. He hasn't been great at staying in touch with his daughter, but now he enjoys using the screen. I can tell it's good for both of them—it strengthens their relationship. I also show Bengt how to find shows and series he likes on his smart TV. I've really gotten good at home tech! My kids even ask *me* for help with the TV now.

Interviewer: *Do you use anything else to stimulate your service users or help them feel calm?*

Anna: Hmm. Yes—with Vera, we use Spotify to play music she loved when she was younger. She can get anxious during morning routines, but when we put on music, we connect better, and she relaxes. She enjoys it when I sing along, too. But it's not just about having a nice moment—I feel like I've gotten better at sensing what my care receivers enjoy and what helps them, like remembering song lyric.

Up-skilling vignette (Home care): Welfare technologies for surveillance and monitoring (e.g. digital safety alarms, cameras, GPS-tracking watches)

Interviewer: *Lisa, you're a nursing assistant in home care services. What was your workday like today?*

Lisa: Well, I always start the morning by checking our central system to see what's happened with "my" service users overnight. Like today, I saw that Elsa had pressed her personal alarm around 3 AM. I'd have to follow up on that when I visited her later. But first, I had to go to someone else. During my first visit of the day, I got a notification on my phone from Arne's GPS watch—he'd left his home. But I could see he was near his house, and he usually hangs out there, so I interpreted that as no cause for concern. Still, those notifications are a bit stressful.

A little later, I got an alert on my phone that a fall sensor had been triggered in Inga's home. Inga always gets so anxious, so I had to leave the service user I was with and rush over to her. When I got there, I saw she'd tripped on a rug edge and was lying in the kitchen. Just as I was helping Inga up and calming her down—thankfully, no classic broken hip or anything—another alert came in from a different service user. I had to rush off without really feeling like I'd finished with Inga. Chaos!

Interviewer: *It sounds like you get interrupted a lot?*

Lisa: Pretty much all the time, to be honest. It wasn't until after lunch that I could go see Elsa—the one who'd triggered the alarm overnight. She was still a bit shaken and afraid of falling out of bed again. So I talked to her a bit about bed rails.

Interviewer: *Did things calm down after that?*

Lisa: Yeah, no—this is how it goes. It's rare *not* to get interrupted. I hardly ever leave a service user feeling like I've done what I needed to. I often have to drop what I'm doing because a GPS alert or fall sensor goes off. I have to shift focus immediately. We colleagues often discuss what happened during a shift to plan better, but there's so much unpredictability that it's impossible. You have to plan for the unplanned, as they say. And then there are all the alerts that force us to drop everything…

De-skilling vignette (Home care): Welfare technologies for surveillance and monitoring (e.g. digital safety alarms, cameras, GPS-tracking watches)

Interviewer: *Lisa, you're a nursing assistant in home care services. What was your workday like today?*

Lisa: Well, I always start the morning by checking our central system to see what's happened with "my" service users overnight. Like today, I saw that Elsa had pressed her personal alarm around 3 AM. I'd have to follow up on that when I visited her later. But first, I had to go to someone else. During my first visit of the day, I got a notification on my phone from Arne's GPS watch—he'd left his home. But I could see he was near his house, and he usually hangs out there. I sent him a message to confirm he was okay. It's actually good that he can go out without one of us having to accompany him. It keeps him active, too.

A little later, I got an alert on my phone that a fall sensor had been triggered in Inga's home. Inga always gets so anxious, so I had to leave the service user I was with and rush over to her. When I got there, I saw she'd tripped on a rug edge in the kitchen. After helping her up and confirming she was okay, we sat down and talked about what we could do to prevent it from happening again. Inga thought it made sense when I suggested removing the kitchen rug so she wouldn't trip on it again.

Interviewer: *Okay, and how did it go with the one who'd triggered the alarm overnight?*

Lisa: Well, after lunch, I went to see Elsa—the one who'd triggered the alarm—so we could talk about what happened. She was still a bit shaken and afraid of falling out of bed again. We discussed different things we could do to make her feel safer, and I could tell she found it reassuring. We talked about installing a bed rail, for example, and she was on board with that.

Interviewer: *Do you and your colleagues usually discuss the day after a shift?*

Lisa: Yes, we share updates to get an overview of our service users' needs and wishes. The different types of technology we have—like alarms, GPS, and so on—actually give us a better overview of the service users, and we can be flexible in how we divide tasks when an alert comes in. Like with Arne—being able to walk around on his own is good for him and for us.

De-skilling Vignette (Nursing home): Mobile ways of working (e.g. documentation apps, digital keys, medication apps)

Interviewer: *Sara, can you tell me how you use your phone during a work shift?*

Sara: Yes… I always start my shift by logging into my smartphone. We have a special app to unlock doors when we visit our residents—no keys needed. Then there's another app where I log and sign off on everything I do with a resident. Oh, and we also have a separate app for signing off on medications. I have to open that one every time I've given someone their medicine.

Interviewer: *Okay. And at the end of your shift, you just log out, close the apps, and you're done? Or…?*

Sara: Well… I also have to fill out some paper forms during the day, so I have to collect those and make sure they end up in the right binder. Then I log into one of the desktop computers at the office and update all the residents' records. Then I'm done.

Interviewer: *How do you find the mobile way of working with your phone?*

Sara: Well… I have all the information I need right there, which is convenient. But I do worry about entering things incorrectly. I'm constantly switching between apps, and I have to stay focused to make sure I'm using the right app for the right task.

I forgot to mention earlier, but when I'm giving medication, for example, I'm standing there with two apps open *at the same time*—and the resident right in front of me. In one app, I verify the correct dosage, and in the other, I sign off that I've given the medication. If the resident starts talking to me at the same time, I easily lose track. It's happened before…

Interviewer: *How do you feel this affects your work?*

Sara: Some residents notice that I get a bit stressed and absorbed in my phone. Some get irritated, while others just go quiet and let me get on with it. So, yeah—it's a lot of nose-in-phone time. Sometimes, when I leave a resident's room, it feels like I've barely made eye contact with them.

It can sometimes feel like the residents just become rows in a checklist that I have to get through during my shift. Like the contact and care for the person diminishes. I end up doing what the phone tells me to do, not necessarily what the resident *needs* that day. It doesn't always feel like warm, personal encounters…

Up-skilling Vignette (Nursing home): Mobile ways of working (e.g. documentation apps, digital keys, medication apps)

Interviewer: *Sara, can you tell me how you use your phone during a work shift?*

Sara: Yes… I always start my shift by logging into my smartphone. It has everything I need for my shift—my tasks and all the information about the residents.

Like yesterday morning, for example. Those of us starting the morning shift saw a warning in the app that Stina, who has chronic arthritis, had had a lot of pain during the night. So we adjusted the schedule so that my first visit in the morning was to her instead of another resident. Before I even went into Stina's room, I knew I should give her pain relief, and then Stina and I could start the day together once she'd had it. We could talk about what she needed next, and so on. And of course, I signed off on the medication in the app right away.

It was a bit like that when I went to see Martin, who has dementia. He's often a bit anxious and confused when you arrive. A colleague had left a note about Martin in the app, describing how he was yesterday. So, on top of seeing what he needs help with at a specific time, the note helped me adjust my approach to how Martin was feeling *that* day. It made it much easier for Martin and me to connect and work together. Afterward, I logged my visit in the app and, if needed, left a note for other colleagues coming after me.

We do this a lot—writing tips for each other about what to keep in mind with different residents. It's really important for handing over to the next colleague or the night shift in a good way.

Interviewer: *Can you give another example of how you use your phone?*

Sara: Yes. When we get new residents on our unit, I sometimes assess what care interventions they need. If I need to do an assessment like that, I get a notification on my phone, and I can prepare using a checklist in the app. So when I meet the new resident, I can work through the checklist and make sure I don't miss anything.

And when I finish my shift, I always go through the interventions I've made with different residents during the day to make sure I haven't forgotten to log anything—and, of course, to sign off on everything.

Interviewer: *How else do you feel this way of working with everything on your phone has affected your work?*

Sara: I feel like I've learned new technology—and actually *improved* my ability to meet residents where they are and provide better care!

Up-skilling Vignette (Nursing home): Welfare technologies for care receiver stimulation and engagement (e.g. robot pets, streaming services)

Interviewer: *Stina, can you tell me about how you work to stimulate the residents using different kinds of technology?*

Stina: Stimulate them? Well, sometimes we gather the residents in the common room. We use the robotic cat Mimmi then. When Mimmi starts purring and moving, the residents usually get happy and talk to each other about their old pets. That also lets me focus on residents who might need extra help or attention.

Mimmi works well with individual residents, too. Yesterday, for example, I brought her to Greta, who was having a tough morning. She was angry and irritated when I tried to help her. But when she held Mimmi and started stroking her, she began purring, and Greta told me about her old cat, Snövit. We had a lovely conversation while we went through her morning routine.

Interviewer: *Do you use any other kinds of technology?*

Stina: Yes! Since the pandemic, we've started using tablets much more. We help the residents video call their families. It's really made it easier for them to stay in touch, especially with relatives who live far away.

Sometimes we have group music sessions. The residents get to choose what they want to listen to, and I—or one of the staff—find it on Spotify on the tablet. Sometimes we even try doing movements to the music.

Interviewer: *How do you feel this has affected your job?*

Stina: I've gotten much better at the technical side—fixing sound, connections, searching for things. I can also help with the smart TV, finding shows or films they like. But I also feel like I've gotten to know the residents in a new way. I've become better at understanding their needs. And sometimes, while the others are occupied—listening to music or petting the cat—I can give extra attention to someone who needs it more.

De-skilling Vignette (Nursing home): Welfare technologies for care receiver stimulation and engagement (e.g. robot pets, streaming services)

Interviewer: *Stina, can you tell me a bit about how you usually work with the robotic cat and the residents?*

Stina: What can I say… After breakfast in the common room, we bring out the robotic cat for the residents. It purrs and moves around. It feels a bit strange sometimes—almost forced. But I don't have time to engage with them then. I have to focus on clearing up and cleaning after breakfast. The cat often malfunctions, too, so I have to rush from the dishes to change the batteries. The residents can get anxious—some think it's a *real* cat dying when it stops working. It's a bit of a hassle.

But at the same time, it *does* let me focus on other tasks. It works sometimes, like with Greta. If she has Mimmi (the cat) in her lap, I can get other necessary work done.

Interviewer: *What other kinds of technology do you use with the residents?*

Stina: Well, these days, residents can video call their families using tablets. But the tech often causes problems, and it takes *my* time to fix it. It's good that they can stay in touch with family, but we end up talking less to each other. It's a bit sad. I don't get to know the residents as well.

Interviewer: *Is there anything else you've noticed about how this affects your work?*

Stina: Hmm, maybe. I feel like I'm becoming more of a technical support service. Less about working *with* the residents. So it's more about emptying the dishwasher, cleaning, measuring out medications… More routine work, less actual care.

De-skilling vignette (Nursing home): Welfare technologies for surveillance and monitoring (e.g. digital safety alarms, cameras, GPS-tracking watches)

Interviewer: *Maria, what was your workday like today?*

Maria: Well, I always start the morning by checking our central system to see if anything in particular happened overnight. Like today, I saw that Elsa had pressed her personal alarm around 3 AM. I'd have to follow up on that later, though I don't always have time. But first, I had to go to someone else. During my first visit, I got a notification on my phone from Arne's GPS watch. He likes to go outside, so he's been given a watch so he can go out on his own. Otherwise, someone would have to accompany him. This time, he'd gone out into the garden. But I could see he was nearby, and it seemed calm, so he could keep walking. Still, the watch pings to let us know where he is. It can be a bit stressful sometimes.

A little later, I got an alert on my phone from the fall sensor in Inga's room. Inga always gets so anxious, so I had to leave the resident I was with and rush over to her. When I got there, I saw she'd tripped on something she'd dropped on the floor and was lying in the middle of the room. Just as I was helping Inga up and calming her down—thankfully, no classic broken hip or anything—another alert came in from a different resident. I had to rush off without really feeling like I'd finished with Inga. Chaos! It's frustrating to have to drop what I'd planned to do.

Interviewer: *It sounds like you get interrupted a lot?*

Maria: Pretty much all the time, to be honest. It wasn't until after lunch that I could take some time to talk to Elsa—the one who'd triggered the alarm overnight. She was still a bit shaken and afraid of falling out of bed again. So I talked to her a bit about bed rails.

Interviewer: *Did things calm down after that?*

Maria: Yeah, no—this is how it goes. It's rare *not* to get interrupted. I hardly ever leave a resident feeling like I've done what I needed to. I often have to drop what I'm doing because a GPS alert or fall sensor goes off. I have to shift focus immediately. We colleagues often discuss what happened during a shift to plan better, but there's so much unpredictability—alarms going off here and there—that it's impossible. You have to plan for the unplanned, as they say. And be ready to run even when we're supposed to be planning or handing over to each other.

Up-skilling vignette (Nursing home): Welfare technologies for surveillance and monitoring (e.g. digital safety alarms, cameras, GPS-tracking watches)

Interviewer: *Maria, what was your workday like today?*

Maria: Well, I always start the morning by checking our central system to see if anything in particular happened overnight. Like today, I saw that Elsa had pressed her personal alarm around 3 AM. I wanted to follow up on that a bit later. But first, I had to start the day with another resident. While I was helping the first resident with their morning routine, I got a notification on my phone from Arne's GPS watch—he'd gone out into the garden. But I could see he was nearby, and I know he enjoys being outside in the mornings. The notification showed everything was calm, so he could keep strolling. Before, someone always had to accompany him, and that didn't always work out, but now we can relax more. It's good that he's getting exercise, too.

A little later, I got an alert on my phone that a fall sensor had been triggered in Inga's room. Inga always gets so anxious, so I had to leave the resident I was with and rush over to her. When I got there, I saw she'd tripped on something and was lying in the middle of the floor. After helping her up and confirming she was okay, we sat down and talked about what we could do to prevent it from happening again. The fall sensor helps us respond faster when someone falls and figure out how to make the room safer. We usually bring up things like this when we meet with colleagues at the end of a shift to hand over.

Interviewer: *Okay, and how did it go with the one who'd triggered the alarm overnight?*

Maria: Well, after the morning routines were done, I could take some time alone with Elsa—the one who'd triggered the alarm—to talk about what happened. She was still a bit shaken and afraid of falling out of bed again. We discussed different things we could do to make her feel safer, and I could tell she found it reassuring. We talked about installing a bed rail, for example, and she was on board with that.

Interviewer: *Do you and your colleagues usually discuss the day after a shift?*

Maria: Yes, we share updates to get an overview of our residents' needs and wishes. Like with the fall sensor, for example. The different types of technology we have—like alarms, GPS, and so on—actually give us a better overview of the residents, and we can be flexible in how we divide tasks when an alert comes in.
